# Supplementary figures and images for: Geo-Temporal Variation in the Antimicrobial Resistance of Escherichia coli in the Community
Source: Antibiotics (Basel). 2025 Feb 25;14(3):233. doi: 10.3390/antibiotics14030233 (PMC11939642; doi:10.3390/antibiotics14030233)

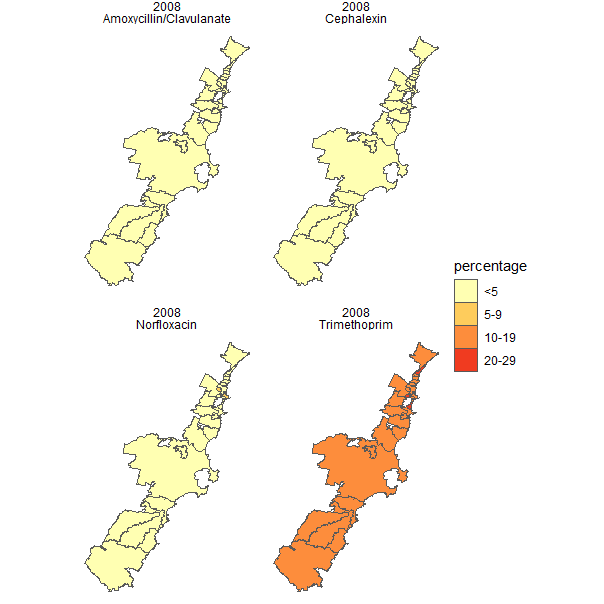

Supplement: Supplementary file 1 [file antibiotics-14-00233-s001.zip › antibiotics-3417995-Video S1.gif]
